# Probability-based nonlinear modeling of neural dynamical systems with point-process inputs and outputs

**DOI:** 10.1186/1471-2202-15-S1-P102

**Published:** 2014-07-21

**Authors:** Roman Sandler, Dong Song, Robert E Hampson, Sam A Deadwyler, Theodore Berger, Vasilis Marmarelis

**Affiliations:** 1 Department of Biomedical Engineering, University of Southern California, Los Angeles, CA 90089, USA; 2 Department of Physiology, Wake Forest School of Medicine, Winston-Salem, NC 27157, USA

## 

One of the great challenges of contemporary neuroscience is understanding in a quantitative manner how neurons and neural ensembles encode and process information in the form of action potentials. The advent of multi-electrode arrays which can record large amounts of data simultaneously from several neurons has made this task more urgent. This task, however, is made difficult by the inherent complexity of neural systems which are highly nonlinear, interconnected, dynamic, and subject to stochastic variations. Furthermore, while several methods exist which offer good predictive performance for spike train modeling, their adoption in the broader neuroscience community has been limited due to their mathematical complexity and lack of interpretability.

Here we present a novel and intuitive methodology of modeling nonlinear dynamic systems with point process inputs and outputs, such as interconnected neuronal ensembles. The method relies on the expression of the nonlinearity in the form of Volterra-like kernels, termed the Probability-Based Volterra (PBV) kernels. The n^th ^order PBV kernel, PBV_n_, is derived in two steps. First, we calculate the conditional probability of an output spike given n input spikes at various lags. Second, we subtract lower order effects to isolate the nth order nonlinearity. Thus, the first PBV kernel, PBV_1_(*τ*), is the conditional probability of an output spike given a spike in the input spike train at time *τ*, minus the probability that there will be a spike in the output, i.e.:

(1) $$PBV_{1}\left(\tau \right)=P\left(y\left[t\right]|x\left[t-\tau \right]\right)-P\left(y\left[t\right]\right)$$

This first order kernel describes a linear impulse response filter similar to that derived from spike triggered averaging and cross-correlation methods. The second order PBV kernel, PBV_2_(*τ_1_, τ_2_*), which is the first nonlinear kernel, is the conditional probability of an output spike given a *pair *of input spikes minus the conditional probability of an output spike given either one of those input spikes individually, i.e.:

(2) $$PBV_{2}\left(\tau_{1},\tau_{2}\right)=P\left(y\left[t\right]\;\text{|}\;x\left[t-\tau_{1}\right]\cap x\left[t-\tau_{2}\right]\right)-P\left(y\left[t\right]|x\left[t-\tau_{1}\right]\right)-P\left(y\left[t\right]|x\left[t-\tau_{2}\right]\right)+P\left(y\left[t\right]\right)$$

This method may be extended to describe the contribution of n pairs spikes to the output in the form of the n^th ^PBV kernel. We show that the PBV kernels are equivalent to the Wiener kernels when the input is a Poisson process, thus placing the PBV kernels in the context of a well-established and rigorous mathematical theory [1].

The proposed PBV methodology was applied to synthetic systems where the ground truth of the model was available. The PBV kernels were found to both accurately estimate the ground truth kernels and to reproduce the given output, thus validating the method. Finally, the proposed PBV methodology was applied to real neural data derived from the CA3 and CA1 regions of the rodent hippocampus [2]. Although here ground truth was not available, the PBV kernels were able to reproduce the output as well as other models which have been validated both mathematically and in-vivo in the context of neural prosthetics [2].
